# Development and Validation of the Early Gastric Carcinoma Prediction Model in Post-Eradication Patients with Intestinal Metaplasia

**DOI:** 10.3390/cancers17132158

**Published:** 2025-06-26

**Authors:** Wulian Lin, Guanpo Zhang, Hong Chen, Weidong Huang, Guilin Xu, Yunmeng Zheng, Chao Gao, Jin Zheng, Dazhou Li, Wen Wang

**Affiliations:** 1 Fuzong Clinical Medical College of Fujian Medical University, Fuzhou 350005, China; 15005974937@163.com (W.L.); zhangguanpo@163.com (G.Z.); ch207789@163.com (H.C.); withdong1996@foxmail.com (W.H.); 13605940092@163.com (G.X.); akisslym@163.com (Y.Z.); wannagc@163.com (C.G.); 15880431386@163.com (J.Z.); 2 Department of Gastroenterology, 900th Hospital of PLA Joint Logistic Support Force, Fuzhou 350025, China

**Keywords:** early gastric cancer, *Helicobacter pylori* eradication, intestinal metaplasia, machine learning, prediction model, endoscopy, xanthoma, map-like redness

## Abstract

**Background**: Gastric cancer (GC) remains a major global health challenge, with rising incidence among patients post-*Helicobacter pylori* (*H. pylori*) eradication, particularly those with persistent intestinal metaplasia (IM). Current risk stratification tools are limited in this high-risk population. **Aim**: To develop, validate, and externally test a machine learning-based prediction model—termed the Early Gastric Cancer Model (EGCM)—for identifying early gastric cancer (EGC) risk in H. pylori-eradicated patients with IM, and to implement it as a web-based clinical tool. **Methods**: This retrospective, dual-center study enrolled 214 H. pylori-eradicated patients with histologically confirmed IM from 900 Hospital and Fujian Provincial People’s Hospital. The dataset was split into a training cohort (70%) and an internal validation cohort (30%), with an external test cohort from the second center. A total of 21 machine learning algorithms were screened using cross-validation and hyperparameter optimization. Boruta and SHAP analyses were employed for feature selection, and the final EGCM was constructed using the top five predictors: atrophy range, xanthoma, map-like redness (MLR), MLR range, and age. Model performance was evaluated via ROC curves, precision–recall curves, calibration plots, and decision curve analysis (DCA), and compared against conventional inflammatory biomarkers such as NLR and PLR. **Results**: The CatBoost algorithm demonstrated the best overall performance, achieving an AUC of 0.743 (95% CI: 0.70–0.80) in internal validation and 0.905 in the external test set. The EGCM exhibited superior discrimination compared to individual inflammatory markers (*p* < 0.01). Calibration analysis confirmed strong agreement between predicted and observed outcomes. DCA showed the EGCM yielded greater net clinical benefit. A web calculator was developed to facilitate clinical application. **Conclusions**: The EGCM is a validated, interpretable, and practical tool for stratifying EGC risk in H. pylori-eradicated IM patients across multiple centers. Its integration into clinical practice could improve surveillance precision and early cancer detection.

## Simple Summary

Gastric cancer is one of the leading causes of cancer deaths worldwide. Although a common stomach bacterium can be treated with medicine, some patients still develop cancer even after treatment. This is especially true for people whose stomach lining has already changed in harmful ways. In this study, we used computer models to analyze medical records and endoscopy images from two hospitals to find patterns that might predict who is more likely to develop early stomach cancer. We created a simple online tool that doctors can use to calculate a patient’s risk. This can help identify high-risk patients earlier and make sure they receive the right follow-up care. Our goal is to improve early detection and save lives through better screening.

## 1. Introduction

Gastric cancer (GC) remains a major global health concern, ranking fifth in cancer incidence and fourth in cancer-related mortality worldwide. In 2020, more than one million new cases were diagnosed, leading to over 769,000 deaths [1,2]. While age-standardized incidence rates are declining in several high-risk regions such as East Asia, demographic transitions forecast a substantial rise in disease burden, with projected increases of 72.2% in incidence and 75.9% in mortality in Asia by 2040 [3,4]. Early detection is pivotal—five-year survival exceeds 90% for early gastric cancer (EGC) but drops below 30% once the disease is advanced [5]. Unfortunately, late diagnosis remains the norm in many settings; for example, in China, approximately 80% of patients are diagnosed at a locally advanced stage [6].

Persistent *Helicobacter pylori* (*H. pylori*) infection is the key driver of GC, initiating a cascade of chronic inflammation, atrophic gastritis, intestinal metaplasia (IM), and neoplasia [7]. The World Health Organization has classified *H. pylori* as a Group I carcinogen since 1994 due to its strong oncogenic potential. Eradication therapy has been shown to significantly reduce the risk of GC, with large-scale randomized trials and meta-analyses reporting a 39% relative risk reduction (RR = 0.61; 95% CI: 0.47–0.79) [8]. Despite this benefit, cancer risk is not eliminated after eradication, especially in patients with pre-existing mucosal damage such as severe atrophy or IM [9]. Furthermore, *H. pylori* eradication is often delayed in clinical practice, leaving ample time for irreversible histological progression to occur before treatment is initiated. Nonetheless, the risk of EGC persists even after successful eradication, particularly in patients with baseline mucosal damage such as atrophy or IM [9]. Surveillance strategies for this growing post-eradication population remain suboptimal, and risk prediction remains poorly defined [10,11].

Although endoscopic screening is the cornerstone of EGC detection, its sensitivity in the post-eradication setting is limited. Conventional white-light imaging often fails to identify subtle premalignant lesions, especially in patients with extensive IM or corpus-predominant atrophy. In a prospective multicenter study, 30.1% of patients developed map-like redness(MLR)—a surrogate for underlying IM—within one year of *H. pylori* eradication, predominantly in the corpus. Importantly, these lesions corresponded to areas of histologic IM that predated eradication, suggesting that endoscopic findings may lag behind histologic progression. High Kyoto classification scores and severe IM were strong predictors of these post-eradication abnormalities (OR = 8.144; 95% CI: 2.811–23.592) [12].

In parallel, machine learning (ML) techniques have been increasingly applied to cancer risk-modeling. A Korean nationwide study involving over 10 million individuals used SHAP-based interpretation to identify key risk factors for gastric cancer; however, model performance remained modest, with AUCs of 0.708 in internal and 0.669 in external validation [13]. Another study employing LASSO-XGBoost achieved high accuracy (AUC = 0.8937), but focused primarily on advanced-stage disease and postoperative survival, limiting its clinical applicability for early detection [14]. Moreover, most existing models rely on coarse clinical features and seldom incorporate endoscopic or histologic markers that are crucial for risk stratification in post-eradication populations.

Given these limitations, we conducted a dual-center retrospective study including patients from both 900 Hospital and Fujian Provincial People’s Hospital, aiming to develop and validate a machine learning-based model for predicting EGC in patients with IM following *H. pylori* eradication. By comparing its performance with conventional inflammatory and nutritional indices, and deploying it as a web-based risk calculator, we sought to improve individualized surveillance and facilitate EGC detection in this vulnerable population.

## 2. Materials and Methods

### 2.1. Study Design and Patient Population

This retrospective cohort study was conducted at two tertiary medical centers in China: the 900th Hospital of the PLA Joint Logistic Support Force and Fujian Provincial People’s Hospital. Clinical and endoscopic data were obtained from institutional endoscopy registries and electronic medical records spanning from January 2019 to December 2024. The study protocol was approved by the Institutional Review Board (IRB Number: Lun Shen Ke 2024-063), and written informed consent was obtained from all participants for the use of their clinical data for research purposes. The study adhered to the principles of the Declaration of Helsinki and followed the TRIPOD (Transparent Reporting of a Multivariable Prediction Model for Individual Prognosis or Diagnosis) guidelines for reporting.

All enrolled patients received a standardized 14-day quadruple therapy regimen for *Helicobacter pylori* eradication. The regimen included esomeprazole 20 mg twice daily, clarithromycin 500 mg twice daily, amoxicillin 1 g twice daily, and colloidal bismuth pectin 200 mg three times daily, administered orally. This protocol is in accordance with current national guidelines for *H. pylori* eradication and has been widely validated in clinical practice. Treatment adherence and post-treatment eradication status were confirmed by a urea breath test performed at least four weeks after therapy completion.

**Figure 1:**
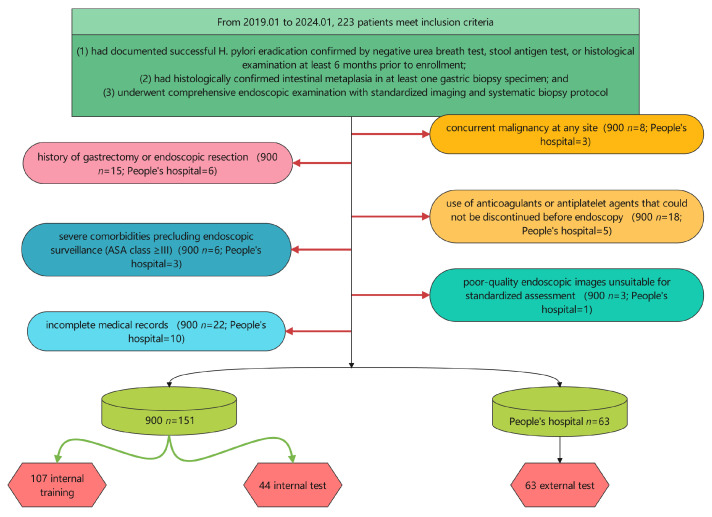
Patient inclusion flow.

Patients were eligible for inclusion if they: (1) had documented successful *H. pylori* eradication confirmed by negative a urea breath test, stool antigen test, or histological examination at least 6 months prior to enrollment; (2) had histologically confirmed intestinal metaplasia in at least one gastric biopsy specimen; and (3) underwent comprehensive endoscopic examination with standardized imaging and systematic biopsy protocol. Exclusion criteria comprised: (1) history of gastrectomy or endoscopic resection; (2) concurrent malignancy at any site; (3) severe comorbidities precluding endoscopic surveillance (ASA class ≥ III); (4) use of anticoagulants or antiplatelet agents that could not be discontinued before endoscopy; (5) incomplete medical records; and (6) poor-quality endoscopic images unsuitable for standardized assessment (Figure 1).

### 2.2. Data Collection

Comprehensive demographic, clinical, endoscopic, and laboratory data were extracted from the institutional electronic medical records system. Two experienced gastroenterologists (Lin Wulian. and Li Dazhou.), each with more than 10 years of experience in advanced endoscopy, independently reviewed all endoscopic images and reports. Any discrepancies were resolved through consensus with a third senior endoscopist (Wang Wen).

### 2.3. Demographic and Clinical Variables

Demographic data included age, sex, height, weight, and calculated body mass index (BMI). Clinical history variables encompassed family history of gastric cancer (first-degree relatives), smoking status, alcohol consumption, and history of *H. pylori* sterilization therapy (regimen and duration documented).

### 2.4. Endoscopic Assessment

All endoscopic examinations were performed using high-definition white-light endoscopy and LCI/ BLI (Fujifilm ELUXEO 7000 (Fujifilm, Tokyo, Japan) or equivalent) by certified endoscopists. The extent of atrophic gastritis was evaluated using the Kimura-Takemoto classification system, which categorizes atrophy into closed-type (C-1, C-2, C-3) and open-type (O-1, O-2, O-3) based on the location of the atrophic border. The atrophy range was then numerically converted (1–6) for statistical analysis, with higher scores indicating more extensive atrophy.

MLR, a characteristic post-eradication finding defined as well-demarcated reddish lesions with irregular margins resembling a geographical map, was documented for presence (yes/no), MLR range (percentage of gastric mucosa affected), and maximum size (in centimeters). The maximum MLR size exceeding 2 cm (maximumMLR2cm) was specifically recorded as a binary variable based on previous literature suggesting its potential predictive value. Xanthoma presence was defined as raised yellowish-white plaques on endoscopic examination. Reflux esophagitis (RE) was graded according to the Los Angeles classification system.

Standardized biopsy protocol followed the Sydney System guidelines with five biopsy sites (antrum greater and lesser curvature, incisura angularis, corpus greater and lesser curvature) plus targeted biopsies of any suspicious lesions. EGC was defined as adenocarcinoma confined to the mucosa or submucosa, irrespective of lymph node status, and was confirmed by two independent pathologists specializing in gastrointestinal malignancies.

### 2.5. Laboratory Parameters and Inflammatory Indices

Blood samples were collected prior to endoscopy. Complete blood count, comprehensive metabolic panel, coagulation profile, and tumor markers were measured using standardized laboratory methods. Specific hematological parameters included neutrophil count, lymphocyte count, platelet count, hemoglobin, albumin level, Absolute monocyte count (AMC), Absolute lymphocyte count (ALC), Red cell distribution wide (RDW), and prothrombin time (PT). Tumor markers were recorded as follows: Carcinoembryonic antigen (CEA), Carbohydrate antigen 19-9 (CA19-9), Carbohydrate antigen 72-4 (CA72-4).

The following inflammatory and nutritional indices were calculated:

1. Neutrophil-to-lymphocyte ratio (NLR) [15] = neutrophil count/lymphocyte count
2. Platelet-to-lymphocyte ratio (PLR) [15] = platelet count/lymphocyte count
3. Lymphocyte-to-monocyte ratio (LMR) [15] = lymphocyte count/monocyte count
4. Prognostic nutritional index (PNI) [15] = 10 × serum albumin (g/dL) + 0.005 × total lymphocyte count (per mm^3^)
5. Systemic immune-inflammation index (SII) [15] = platelet count × neutrophil count/lymphocyte count
6. Systemic inflammation response index (SIRI) [15] = neutrophil count × monocyte count/lymphocyte count
7. Geriatric nutritional risk index (GNRI) [15] = 1.489 × albumin (g/L) + 41.7 × (weight/ideal weight)
8. Hemoglobin, albumin, lymphocyte, and platelet score (HALP) [16] = hemoglobin (g/L) × albumin (g/L) × lymphocyte count/platelet count
9. Platelet-to-albumin ratio (PAR) [16] = platelet count/serum albumin (g/L)

These indices were selected based on previous literature demonstrating their association with inflammation, nutritional status, and cancer risk in upper gastrointestinal disorders. All indices were calculated using laboratory values obtained during the same sampling period to ensure internal consistency.

### 2.6. Feature Selection and Engineering

Feature selection was performed using a multi-stage approach to identify the most predictive variables while minimizing multicollinearity. Initially, univariate analysis assessed the association between each potential predictor and the presence of EGC. Variables with *p* < 0.1 in univariate analysis were considered for further evaluation.

Correlation analysis was performed using Spearman’s rank correlation coefficient for continuous variables, with pairs showing |*r*| > 0.7 considered highly correlated. For highly correlated feature pairs, the variable with stronger univariate association with the outcome was retained. Boruta was then applied to identify the optimal feature subset, using the area under the receiver operating characteristic curve (AUC-ROC) as the performance metric.

Additionally, SHapley Additive exPlanations (SHAP) values were calculated to quantify the contribution of each selected feature to the model output. This approach allowed us to rank features based on their absolute SHAP values and select the optimal feature subset.

### 2.7. Model Development and Validation

The dataset was randomly split into training (70%) and validation (30%) sets, stratified by the presence of EGC to maintain class distribution. To address potential class imbalance, we employed the Synthetic Minority Over-sampling Technique (SMOTE) on the training set only, creating synthetic instances of the minority class to achieve balanced class distribution.

We systematically evaluated 21 ML algorithms with varying computational approaches and complexity levels, including:

- Tree-based methods: CatBoost, LightGBM, Random Forest, Extra Trees, Gradient Boosting, Decision Tree
- Ensemble methods: Bagging, AdaBoost
- Support vector machines: SVC (Polynomial kernel), SVC (Radial Basis Function kernel), Linear SVC
- Bayesian methods: Gaussian Naive Bayes, Bernoulli Naive Bayes
- Linear models: Quadratic Discriminant Analysis (QDA), Linear Discriminant Analysis (LDA), Ridge Classifier, Logistic Regression, Stochastic Gradient Descent (SGD) Classifier
- Neural networks: Multi-Layer Perceptron (MLP)
- Instance-based methods: K-Nearest Neighbors (*k* = 3), K-Nearest Neighbors (*k* = 5)

Hyperparameter optimization was conducted using Bayesian optimization with five-fold cross-validation, allowing 100 iterations to identify optimal parameter configurations for each algorithm. The search space for hyperparameters was defined based on established literature and computational constraints.

Model performance was evaluated using the AUC-ROC, sensitivity, specificity, positive predictive value (PPV), negative predictive value (NPV), and accuracy. Additionally, the area under the precision–recall curve (AUC-PR) was calculated to account for potential class imbalance. Confidence intervals (95% CI) for all metrics were derived using 1000 bootstrap resamples.

### 2.8. Model Comparison and Ensemble Creation

The best-performing algorithm based on validation set AUC-ROC was selected as the primary prediction model, designated as the Early Gastric Cancer Model (EGCM). For comprehensive comparison, we developed nine simplified models based on established inflammatory and nutritional indices: (1) NLR model, (2) PNI model, (3) PLR model, (4) SII model, (5) SIRI model, (6) GNRI model, (7) HALP model, (8) LMR model, and (9) PAR model. These comparisons were performed using DeLong’s test for correlated ROC curves.

### 2.9. Calibration and Decision Curve Analysis

Model calibration was assessed using calibration plots, comparing predicted probabilities against observed event rates across deciles of predicted risk. The Hosmer–Lemeshow test and Brier score were calculated to quantify calibration quality. Additionally, decision curve analysis (DCA) was performed to evaluate the clinical utility of the model across a range of decision thresholds, measuring the net benefit of using the model compared to strategies of treating all patients or no patients.

### 2.10. Interpretability Analysis

To enhance clinical interpretability. SHAP summary and dependency plots were created to illustrate both global and local feature importance.

### 2.11. Web-Based Calculator Development

A user-friendly web-based calculator was developed using the Flask framework (Python) with a responsive HTML frontend. The application was designed to accept input of the key predictive variables identified by the model, process them using the pre-trained prediction algorithm, and return the estimated probability of EGC. The calculator incorporates data validation, normalization according to the training dataset parameters, and visualization of individual risk factors. The web application was deployed on a secure server with SSL encryption to ensure data privacy and underwent usability testing with a panel of five experienced gastroenterologists.

### 2.12. Statistical Analysis

All statistical analyses were performed using R version 4.2.0 (R Foundation for Statistical Computing, Vienna, Austria) and Python 3.9 with scikit-learn 1.0.2, XGBoost 1.5.1, and SHAP 0.40.0 packages. Continuous variables were reported as mean ± standard deviation or median (interquartile range) based on distribution normality assessed by the Shapiro–Wilk test. Categorical variables were presented as counts and percentages. Between-group comparisons were conducted using Student’s *t*-test or Mann–Whitney U test for continuous variables and the chi-square test or Fisher’s exact test for categorical variables, as appropriate.

For comparative analysis of inflammatory indices, we constructed receiver operating characteristic (ROC) curves for each index and calculated the corresponding AUC values with 95% confidence intervals. Pairwise comparisons of AUC values were performed using DeLong’s method with Bonferroni correction for multiple comparisons.

All statistical tests were two-sided, with *p* < 0.05 considered statistically significant. *p*-values were adjusted for multiple comparisons using the Benjamini–Hochberg procedure to control the false discovery rate.

## 3. Result

### 3.1. Basic Information of All Patients

A total of 214 patients were included in this multicenter cohort, comprising 126 non-EGC and 88 EGC cases. The dataset was divided into internal training (*n* = 107), internal testing (*n* = 44), and external testing (*n* = 63) sets. Across all cohorts, 77.1% were male, and the median age was 62 years. Key endoscopic features such as map-like redness (MLR) and xanthoma were observed in 61.7% and 35% of patients, respectively. The atrophy range had a median value of 3 [IQR: 2–4], while the MLR range was 2 [IQR: 0–3]. The median *H. pylori* eradication time was 3 years [IQR: 2–7]. There were no statistically significant differences (all *p* > 0.05) among the internal training, internal testing, and external testing cohorts in any demographic, endoscopic, or laboratory variables, indicating comparability across the datasets. Full variable distributions are detailed in Table 1.

### 3.2. Information in Training Set

In our analysis of variables associated with EGC in the training set (Table 2), several factors demonstrated statistically significant differences between affected and non-affected patients. Demographically, male patients exhibited a significantly higher prevalence of EGC compared to females (88.9% vs. 11.1%, *p* = 0.047), and affected individuals were notably older (66.71 ± 7.64 vs. 56.69 ± 10.48 years, *p* < 0.001). Endoscopic findings revealed particularly strong associations, with MLR present in 97.8% of EGC cases versus 40.3% in the non-cancer group (*p* < 0.001), and MLR exceeding 2 cm diameter more frequently observed in the cancer group (57.8% vs. 22.6%, *p* < 0.001). Similarly, xanthoma was significantly more prevalent among EGC patients (66.7% vs. 16.1%, *p* < 0.001), while quantitative endoscopic parameters including atrophy range (4.29 ± 1.16 vs. 2.53 ± 0.86, *p* < 0.001) and MLR range (2.93 ± 1.23 vs. 1.02 ± 1.29, *p* < 0.001) were markedly elevated. These findings suggest that specific clinical, endoscopic, and serological parameters may serve as valuable indicators for EGC detection in clinical practice. In internal training cohorts, we recorded the time interval between *H. pylori* eradication and the date of endoscopic diagnosis, referred to as eradication time. Comparative analysis revealed no statistically significant difference in eradication time between the EGC and non-EGC groups (*t p* = 0.057), suggesting that eradication duration alone may not be a strong predictor of early gastric cancer in this selected population.

### 3.3. Machine Learning Model Performance in EGC Discrimination

**Figure 2:**
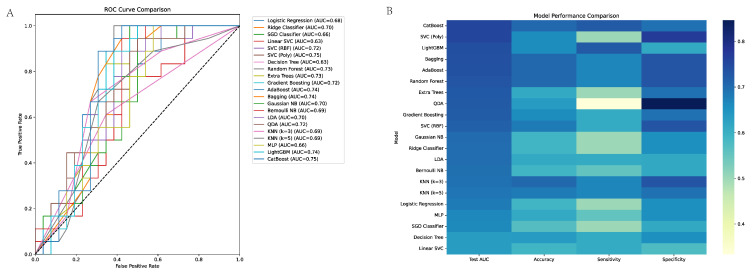
Model performance comparison in different machine learning (**A**): Comparison of Performance of Different Machine Learning Models (Based on Test AUC) Models like CatBoost, SVC (Poly), and LightGBM have an AUC value close to 0.8, showing excellent performance, while models such as SGD Classifier have an AUC of around −0.4, with relatively poor performance. (**B**): Comparison of Performance of Different Machine Learning Models (Based on Accuracy, Sensitivity, and Specificity).

In our evaluation of ML models for EGC prediction, the top-performing algorithms demonstrated moderate to good discriminative ability. Among the 21 models tested, CatBoost achieved the highest predictive performance with identical test AUCs of 0.754. LightGBM ranked third with an AUC of 0.741 and sensitivity matching CatBoost (0.722) but with lower specificity (0.615). Bagging and AdaBoost classifiers followed closely with AUCs of 0.739 and 0.737, respectively, both achieving identical performance metrics (accuracy: 0.704, sensitivity: 0.666, specificity: 0.730, PPV: 0.631, NPV: 0.76). Notably, while CatBoost demonstrated the best balance between sensitivity and specificity, ensemble methods generally outperformed individual classifiers, suggesting that combining multiple algorithms may enhance the predictive capability for EGC detection. The relatively modest AUC values across all models indicate that further refinement of feature selection and algorithm optimization may be necessary for clinical implementation (Showed as Table 3 and Figure 2).

### 3.4. Feature Selection

**Figure 3:**
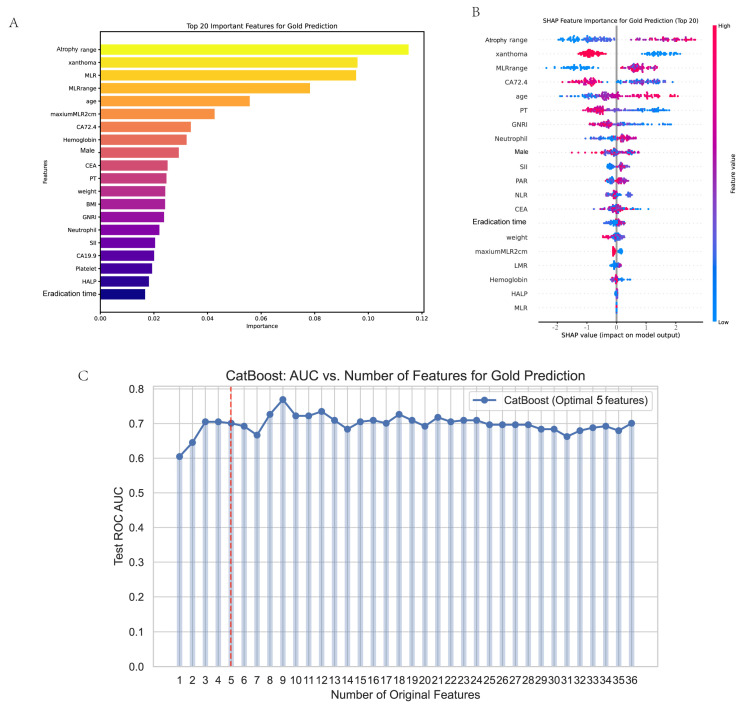
Feature selection process. (**A**) The “Top 20 Important Features for Gold Prediction. (**B**) The “SHAP Feature Importance for Gold Prediction (Top 20)” utilizes SHAP values to illustrate the influence of each feature on the model output. Positive SHAP values imply a positive influence, while negative ones indicate a negative influence. This enables researchers to zero in on key features. (**C**) The “CatBoost: AUC vs. Number of Features for Gold Prediction” depicts the variation in the AUC value in the CatBoost model as the number of original features changes. Initially, augmenting the number of features enhances the AUC, but beyond a certain point, the improvement plateaus. This figure offers multi-faceted insights for optimizing prediction models in gold-standard-prediction research.

**Figure 4:**
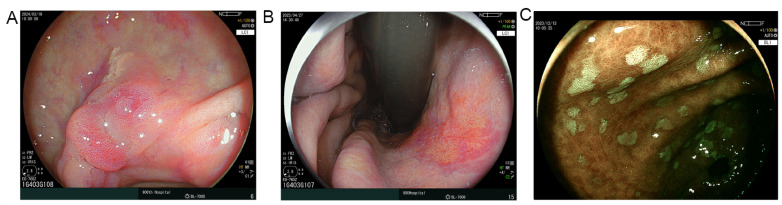
MLR and xanthoma. (**A**): A 72-year-old man with map-like redness on the lesser curvature of the distal antrum; a yellowish nodule was observed adjacent to the lesion. Histopathology confirmed the presence of early gastric cancer. (**B**): A 68-year-old man with map-like redness identified at the lesser curvature of gastric body. Histopathology confirmed the presence of early gastric cancer. (**C**): A 70-year-old man with multiple flat elevated lesions in the distal antrum consistent with intestinal metaplasia; no map-like redness was observed. Histopathology confirmed the absence of early gastric cancer.

Variable importance was assessed using the Boruta algorithm on the top 20 ranked variables. The ten most important variables (atrophy range, xanthoma, MLR, MLR range, age, maximum MLR > 2 cm, CA72–4, Hemoglobin, male, CEA) identified are depicted in Figure 3A. Furthermore, SHAP analysis was performed to visualize the contribution of each variable to the model’s output (Figure 3B). To determine the optimal number of variables for model construction, we evaluated the AUC values across models with varying numbers of predictors (Figure 3C). Although the model including nine variables exhibited a slightly higher AUC, we ultimately selected a five-variable model comprising atrophy range, xanthoma, MLR, MLR range, and age. This decision was based on a balance between predictive performance and clinical applicability. The five-variable model achieved satisfactory discriminative power while maintaining parsimony and interpretability, which are essential for real-world implementation. These variables were also consistently ranked among the most important features by both Boruta and SHAP analyses. Therefore, the final predictive model for EGC—termed the Early Gastric Cancer Model (EGCM)—was constructed using these five features. Representative endoscopic findings for MLR and xanthoma are illustrated in Figure 4.

### 3.5. ROC

**Figure 5:**
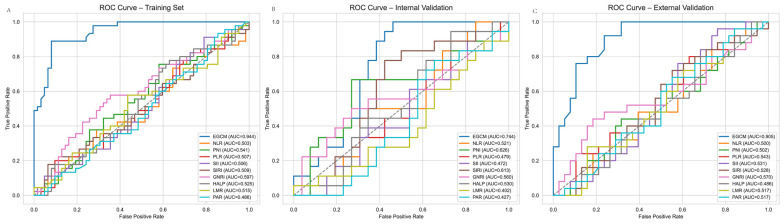
ROC curve (**A**) Internal training set. (**B**) Internal validation set. (**C**) External validation set.

To evaluate the diagnostic performance of the proposed EGCM, we compared its classification metrics with those of conventional inflammation- and nutrition-based indices across the training, internal validation, and external validation cohorts (Table 4). In the training set, EGCM demonstrated outstanding discrimination with an AUC of 0.943 (95% CI: 0.900–0.987), sensitivity of 0.888, specificity of 0.919, and accuracy of 0.906. In contrast, all comparative indices (NLR, PLR, SII, SIRI, LMR, PAR) exhibited poor performance, with AUCs close to 0.5, sensitivities of 0, and specificities of 1.0, reflecting high false-negative rates. Among them, GNRI performed relatively better (AUC: 0.597; 95% CI: 0.504–0.690), albeit with a sensitivity of only 0.155. In the internal test set, EGCM retained strong performance, achieving an AUC of 0.743 (95% CI: 0.614–0.872), with sensitivity, specificity, and accuracy of 0.555, 0.692, and 0.636, respectively. Among traditional indices, only PNI (AUC: 0.626), SIRI (AUC: 0.613), and GNRI (AUC: 0.559) showed marginal predictive capacity, whereas the remaining models had AUCs < 0.53 and zero sensitivity. Importantly, in the external validation cohort (Fujian Provincial People’s Hospital), EGCM achieved a high AUC of 0.905 (95% CI: 0.832–0.977), with sensitivity of 0.76, specificity of 0.894, and accuracy of 0.841, demonstrating strong generalizability across independent populations. In contrast, all traditional indices failed to exceed AUCs of 0.57, and again showed poor sensitivity (0 for most). These results, visualized in Figure 5, underscore the superior discriminative ability and clinical utility of the EGCM compared to established indices across multiple datasets.

### 3.6. PR Curve

**Figure 6:**
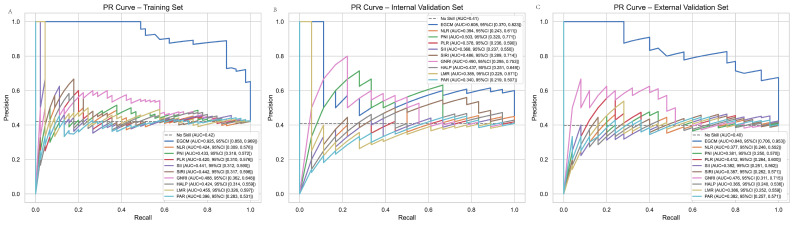
PR curve (**A**) Internal training set. (**B**) Internal validation set. (**C**) External validation set.

To further evaluate the discriminative performance of the EGCM, precision–recall (PR) curves were analyzed across the training, internal validation, and external validation cohorts (Figure 6A–C). In the training set (Figure 6A), the EGCM demonstrated excellent classification performance, characterized by a high and well-shaped PR curve. This reflects the model’s strong ability to maintain high precision while achieving robust recall in identifying early gastric cancer (EGC) cases. In comparison, all conventional inflammation- and nutrition-based indices (e.g., NLR, PLR, SII, SIRI, GNRI, PNI, LMR, HALP, PAR) showed PR curves that remained close to the baseline, indicating limited ability to detect true positives and a tendency toward high false-negative rates. In the internal validation set (Figure 6B), the EGCM maintained a markedly superior PR curve, though with a moderate attenuation in performance compared to the training set—an expected outcome during validation. The model still achieved a favorable balance between precision and recall, supporting its reliability and generalizability within the same institutional cohort. Crucially, in the external validation cohort (Figure 6C), the EGCM retained notably better discriminative performance than all other indices. The PR curve remained well above those of traditional models, reaffirming the EGCM’s robust predictive utility in an independent patient population. Conventional indices, once again, failed to contribute meaningfully, with their PR curves exhibiting near-baseline performance.

### 3.7. Calibration Curve

**Figure 7:**
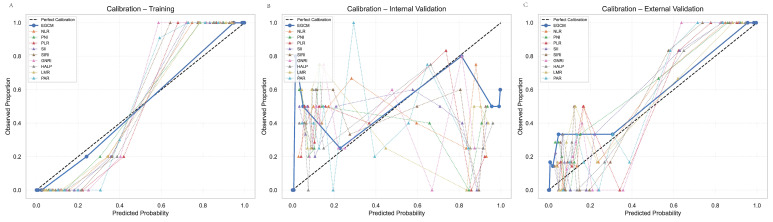
Calibration curve (**A**) Internal training set. (**B**) Internal validation set. (**C**) External validation set.

In addition to discrimination, calibration analysis was performed to evaluate the agreement between predicted probabilities and observed outcomes across three datasets. As shown in Figure 7 and Table 5, the EGCM exhibited excellent calibration in both the internal and external validation cohorts. In the training set, EGCM achieved an exceptionally low Brier score of 0.001, indicating minimal prediction error, and a Hosmer–Lemeshow (HL) *p*-value of 0.999, suggesting an excellent goodness-of-fit. The calibration curve (Figure 7A) closely aligned with the 45-degree reference line, visually confirming the strong concordance between predicted and actual risks. In the internal test set, the model maintained good calibration performance with a Brier score of 0.002 and a non-significant HL *p*-value of 0.261, supporting its generalizability within the same center. The calibration plot (Figure 7B) showed consistent alignment with the ideal line. Importantly, the EGCM also demonstrated reliable performance in the external test cohort, with a Brier score of 0.038 and HL *p*-value of 0.285, indicating acceptable calibration even in an independent patient population. The calibration plot in Figure 7C confirmed the model’s robustness across centers. In contrast, all comparative indices—including NLR, PNI, PLR, SII, SIRI, GNRI, HALP, LMR, and PAR—exhibited significantly worse calibration metrics. These models had substantially higher Brier scores (up to 0.461) and statistically significant HL *p*-values (<0.001) in both internal and external validation, indicating poor predictive reliability. Their calibration curves deviated markedly from the reference line, reflecting poor agreement between predicted and actual risks.

### 3.8. DCA

**Figure 8:**
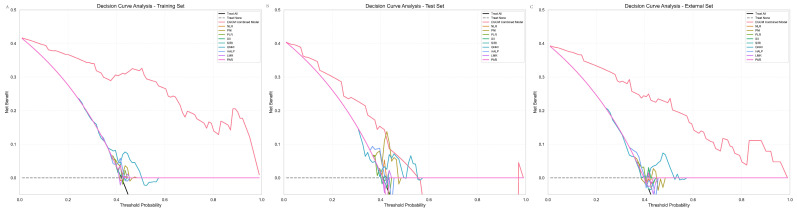
DCA curve (**A**) Internal training set. (**B**) Internal validation set. (**C**) External validation set.

To assess the clinical utility of the Early Gastric Cancer Model (EGCM), we performed decision curve analysis (DCA) alongside reclassification metrics across three cohorts: internal training, internal validation, and external validation sets. As shown in Figure 8 and Table 6, the EGCM consistently demonstrated the highest net clinical benefit across a wide range of threshold probabilities in all datasets. In the training cohort (Figure 8A), EGCM outperformed all conventional inflammatory and nutritional indices, including NLR, PNI, PLR, SII, SIRI, GNRI, HALP, LMR, and PAR. The DCA curve of EGCM remained distinctly above both the “treat-all” and “treat-none” strategies. Quantitatively, all comparator models showed significantly negative NRI values (e.g., NLR: −0.808, PNI: −0.808) and IDI values (e.g., GNRI: −0.571, HALP: −0.590) with *p*-values < 0.001, indicating inferior reclassification and discrimination performance. In the internal validation cohort (Figure 8B), EGCM again exhibited superior net benefit compared to other models, although the performance margin narrowed. All indices showed consistently negative IDI values (range: −0.243 to −0.263), and most NRI values were also negative, confirming suboptimal risk classification (e.g., PLR: NRI = −0.247, *p* = 0.0768). Notably, in the external validation cohort, EGCM maintained its clinical advantage. As shown in Figure 8C, its DCA curve remained highest across clinically relevant thresholds. All alternative indices showed significantly negative NRI values (e.g., NLR: −0.654, PNI: −0.654) and IDI values (e.g., GNRI: −0.460, HALP: −0.478) with *p* < 0.001, reaffirming the consistent superiority of EGCM in external testing.

### 3.9. Presentation of Various Predictive Scenarios

**Figure 9:**
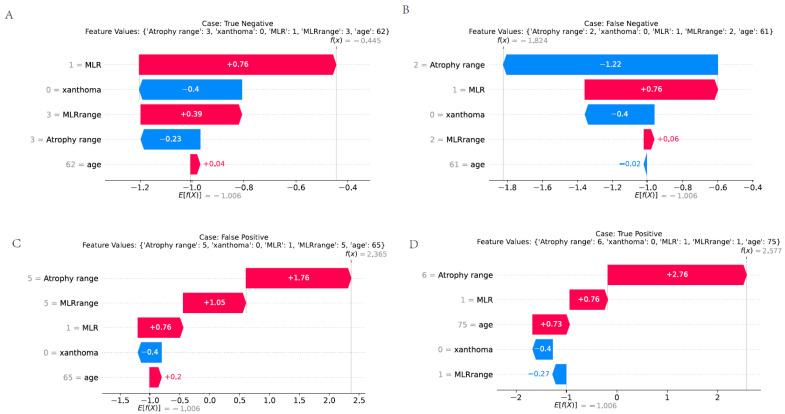
Illustrates different patient cases in the context of a predictive model. (**A**) True negative. (**B**) False negative. (**C**) False positive. (**D**) True positive.

Figure 9 presents a detailed analysis of different patient cases using feature values and model outputs, offering insights into the performance of a predictive model in the context of EGC. Each sub-figure represents a distinct patient scenario, highlighting the relationship between feature values and the model’s predicted outcome. In Figure 9A, we have a True Negative case. The patient’s feature values are provided, such as “range:3”, “xanthoma:0”, “MLR:1”, “MLRrange:3”, and “age:62”. The model output *f*(*x*) = −0.445, and the equation for calculating the model output seems to be a weighted sum of feature values, for example, terms like “1 = MLR + 0.76”, “3 = MLRrange + 0.39”. The expected value *E*[*f*(*X*)] = −1.006, and the relatively negative value of *f*(*x*) indicates that the model correctly predicts this case as negative, aligning with the actual outcome. Figure 9B shows a False Negative case. Here, with feature values like “range’:2”, “xanthoma’ 0”, “MLR:1”, “MLRrange:2”, and “age:61”, the model output *f*(*x*) = −1.824. Although the model classifies this as a negative case, it is actually a positive case in reality. The large negative value of *f*(*x*) compared to the True Negative case in sub-figure A might be due to the combination of feature values, suggesting that the model fails to accurately identify this positive case. Figure 9C represents a False Positive case. The patient has feature values “range: 5”, “anthoma”: 0”, “MLR:1”, “MLRrange:5”, and “age:65”, and the model output *f*(*x*) = 2.365. The positive value of *f*(*x*) leads the model to predict this as a positive case, while it is actually negative. The large positive value of *f*(*x*) could be a result of the specific combination of feature values, causing the model to misclassify. Figure 9D is another True Negative case with feature values “range:3”, “xanthoma: 0”, “MLR: 1”, “MLRrange’:3”, and “age:62”, similar to sub-figure A. The model output *f*(*x*) = −0.445 and *E*[*f*(*X*)] = −1.006, correctly indicating a negative outcome. Overall, Figure 8 visually demonstrates how the model performs differently for various patient cases based on their feature values. The False Negative and False Positive cases highlight potential areas where the model can be improved, while the True Negative cases show the model’s correct classification ability. Understanding these differences can help in refining the predictive model for more accurate EGC diagnosis.

### 3.10. Web Calculate

**Figure 10:**
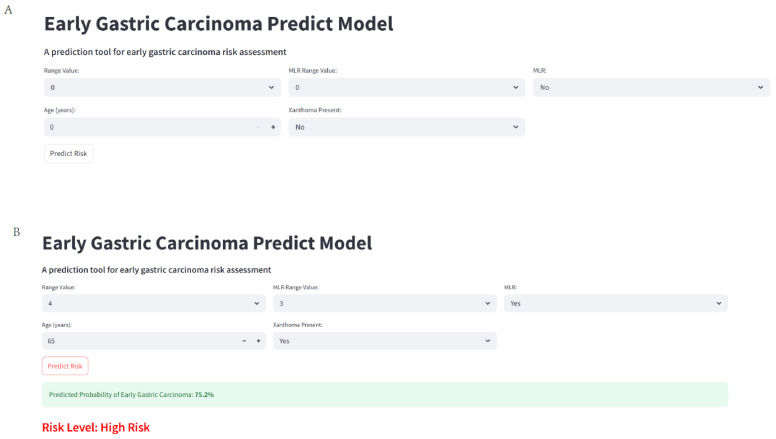
Web-based calculator for early gastric carcinoma risk assessment. (**A**) displays the initial state of the calculator with default input values. (**B**) shows the outcome after entering specific values, including a predicted probability of early gastric carcinoma (75.2% in this case) and a corresponding risk level (High Risk).

In the realm of early gastric carcinoma research, a novel web-based calculator has been developed to streamline risk assessment, and its interface is presented in Figure 10. This calculator, accessible at https://ktdi3dqqj68uwpu4x9odw9.streamlit.app/ (accessed on 2 June 2025), offers a user-friendly platform for estimating the probability of early gastric carcinoma. Figure 10A shows the default view of the calculator. It features input fields for “Range Value”, “MLR Range Value”, “MLR”, “Age (years)”, and “Xanthoma Present”. Initially, these fields are set to default values like 0 for numerical inputs and “No” for the “Xanthoma Present” option. This clean and intuitive starting state allows users, potentially medical professionals or researchers, to easily input patient-specific data. Upon inputting relevant patient information, as demonstrated in Figure 10B, the calculator generates a risk prediction. For instance, when “MLR” is set to 3, “Xanthoma Present” is toggled to “Yes”, and the “Age (years)” is entered as 65, the calculator computes a predicted probability of early gastric carcinoma. In this example, the result indicates a 75.2% probability, categorizing the patient as being at “High Risk”.

## 4. Discussion

In this multicenter retrospective study involving H. pylori-eradicated patients with histologically confirmed IM from two independent institutions, we developed and externally validated a robust machine learning-based predictive model—EGCM—using the CatBoost algorithm. Based on comprehensive feature selection incorporating Boruta and SHAP analyses, five clinically accessible predictors were identified: atrophy range, xanthoma, MLR, MLR range, and age. The EGCM demonstrated consistently strong diagnostic performance, achieving an AUC of 0.743 in the internal validation cohort and 0.905 in the external validation cohort, along with excellent calibration and superior clinical net benefit compared to traditional inflammation- and nutrition-based indices. To facilitate clinical implementation, the model was deployed as a user-friendly online risk calculator, offering individualized risk estimation to optimize surveillance strategies for EGC in this high-risk population.

Gastric mucosal atrophy emerged as a central risk factor for EGC, consistent with previous findings. Adachi et al. demonstrated that greater endoscopic atrophy significantly predicted GC risk in post-eradication patients [17]. Similarly, Kuraoka et al. reported that patients with severe atrophy and no prior eradication had a higher frequency of elevated-type GC, supporting the concept of a persistent carcinogenic field [18].

Among endoscopic features, MLR showed a particularly strong association with EGC risk. Matsumoto et al. found MLR in 25.3% of patients one year post-eradication, with higher odds in those with IM (OR = 2.794, 95% CI: 1.155–6.757) and acid inhibitor use (OR = 1.948, 95% CI: 1.070–3.547); MLR itself was associated with GC (OR = 2.432, 95% CI: 1.264–4.679) [19]. In a multicenter prospective study, the MLR rate reached 30.1%, and all patients with MLR had pre-existing IM at corresponding sites; IM remained significantly associated with MLR (OR = 8.144, 95% CI: 2.811–23.592) [12]. Our MLR incidence (20%) was slightly lower, likely due to shorter surveillance intervals or lower baseline mucosal severity. Crucially, unlike previous binary MLR classifications, our model quantifies the MLR extent, which may explain its stronger predictive power.

Long-term data reinforce MLR’s carcinogenic significance. Iwata et al. observed increasing MLR prevalence from 3.6% to 18.7% over 15 years (*p* = 0.03) [9], highlighting the need for prolonged surveillance. Moreover, MLR-associated EGCs often present as reddish depressed lesions. Tahara et al. reported magnifying endoscopy with narrow-band imaging (ME-NBI) achieved 93.9% diagnostic accuracy in distinguishing neoplastic from benign lesions [20].

GX also emerged as a significant endoscopic biomarker. Shen et al. identified GX as an independent risk factor for both precancerous lesions (OR = 3.197, 95% CI: 2.791–3.662) and gastric cancer (OR = 1.794, 95% CI: 1.394–2.309) [21]. Gao et al. found higher GX prevalence in patients with precancerous lesions (14.9%) and GC (19.8%) compared to chronic gastritis (6.2%) [22]. Feng et al. further demonstrated that GX correlates with atrophic gastritis (OR = 1.83), IM (OR = 2.42), and *H. pylori* infection (OR = 1.32); multiple GXs were associated with a higher burden of precancerous changes, suggesting a dose-dependent relationship [23].

Age was another independent predictor of EGC. Our findings of increasing age-related risk align with those of Iwata et al. [9] and Wei et al. [24], who reported that severe atrophy (OR = 2.71) and IM (OR = 5.0, *p* < 0.001) predicted post-eradication EGC. Adachi et al. noted that 47.5% of elderly patients showed no regression of atrophy after eradication [17], underscoring persistent mucosal risk. Matsushima et al. found that individuals ≥80 years old accounted for 50% of GC-related deaths despite eradication, emphasizing the need for vigilant endoscopic follow-up in this population [25]. Conversely, Jung et al. reported reduced GC incidence in patients ≥ 70 after eradication (SIR = 0.56, 95% CI: 0.52–0.61), though their study focused on primary prevention [26]. The steeper risk gradient in our cohort may reflect inclusion of patients with baseline metaplasia undergoing post-eradication surveillance.

The EGCM outperformed traditional biomarkers such as NLR, which, although statistically associated with GC (OR = 1.38, 95% CI: 1.04–1.83) [17], offers limited predictive utility alone. By integrating demographic, histologic, and endoscopic parameters—particularly MLR range and GX—the EGCM reflects the multifactorial nature of gastric carcinogenesis. The inclusion of corpus-predominant atrophy and advanced endoscopic signs aligns with recent data demonstrating their superior prognostic value [27,28].

In-depth analysis of model outputs, as illustrated in Figure 9, revealed the presence of both false positive and false negative cases, underscoring the need for continuous refinement of the EGCM. False negatives are particularly concerning in the context of EGC surveillance, as they may lead to missed opportunities for early intervention. For example, in Figure 9B, despite moderate-risk feature values, the model underestimated the malignancy risk, highlighting the limitations of current feature representation in capturing subtle disease signals. Conversely, false positives, such as in Figure 9C, may result in unnecessary anxiety or invasive procedures for low-risk individuals. These misclassifications suggest that the model, while robust overall, may benefit from incorporating additional predictive dimensions—such as mucosal texture, immune status, or metabolic profiles—to enhance its discriminatory power. Future iterations of the model should prioritize reducing these critical errors through expanded datasets, inclusion of multimodal biomarkers, and ongoing prospective validation in diverse populations.

## 5. Limitations

Several limitations warrant consideration. The retrospective, single-center design may limit generalizability and introduce selection bias. Although expert endoscopists performed consensus readings, interobserver variability in assessing atrophy, xanthoma, and MLR could affect reproducibility. We lacked an external validation set from an independent institution; thus, EGCM’s performance in diverse ethnic and geographic populations remains to be confirmed. Finally, unmeasured factors—such as dietary patterns, genetic polymorphisms, and microbiome alterations—may further modulate EGC risk but were not captured in our dataset.

## 6. Future Directions

Prospective, multicenter validation of EGCM is essential to establish its generalizability and clinical utility across varying practice settings. Incorporation of advanced imaging modalities (e.g., narrow-band imaging, confocal laser endomicroscopy) and emerging molecular biomarkers (e.g., DNA methylation signatures [29], microRNA profiles [30]) could augment model precision [31]. Additionally, longitudinal studies assessing dynamic risk changes with repeat endoscopy and laboratory assessments will clarify the optimal surveillance cadence [32,33]. Finally, health economic analyses should evaluate the cost-effectiveness of EGCM-guided surveillance pathways compared to standard care.

Despite the promising performance of our EGCM, we acknowledge that direct comparison with previously published machine learning (ML) models for early gastric cancer (EGC) prediction remains limited. This is primarily due to differences in target populations, model endpoints, and availability of variables. Most existing ML-based gastric cancer models have focused on advanced-stage disease, postoperative outcomes, or general cancer risk stratification, often relying on features such as imaging, genomic profiles, or metabolic panels that were not available in our dataset.

Furthermore, recent studies have highlighted the potential value of integrating metabolic and immune system biomarkers—including cytokines, chemokines, microbiome signatures, and host gene expression—in gastric cancer risk prediction [34,35]. These multimodal biological indicators could help capture the tumor microenvironment and systemic host responses more accurately. Future studies should therefore explore the integration of these multi-omic and systemic inflammatory features into ML-based prediction tools to enhance accuracy and biological interpretability, especially for the post-*H. pylori* eradication population.

## 7. Conclusions

We developed and externally validated a robust, interpretable machine learning model—Early Gastric Cancer Model (EGCM)—to predict the risk of EGC in H. pylori-eradicated patients with IM. Using data from two independent medical centers, EGCM was built upon five accessible clinical and endoscopic features: atrophy range, xanthoma, MLR, MLR range, and age. The model demonstrated superior discrimination and calibration compared to conventional inflammatory and nutritional indices across both internal and external cohorts. Decision curve analysis confirmed its clinical utility, and a web-based calculator was deployed to facilitate individualized risk estimation. EGCM provides a practical tool for identifying high-risk individuals, potentially guiding more effective surveillance strategies and improving early detection outcomes. While our current model has shown promising performance, future studies should aim to directly or indirectly compare EGCM with other published machine learning-based prediction tools for EGC. In addition, incorporating metabolic, immunologic, and multi-omic data could further enhance the model’s predictive accuracy, biological interpretability, and clinical applicability, ultimately contributing to more effective risk stratification and personalized surveillance strategies.

## Figures and Tables

**Table 1:** Basic information in all patients.

Variable	Total	Internal Train	Internal Test	External Test	*p*_Value
No early gastric carcinoma	126 (58.9%)	62 (57.9%)	26 (59.1%)	38 (60.3%)	0.95
Early gastric carcinoma	88 (41.1%)	45 (42.1%)	18 (40.9%)	25 (39.7%)	
Female	49 (22.9%)	23 (21.5%)	14 (31.8%)	12 (19%)	0.27
Male	165 (77.1%)	84 (78.5%)	30 (68.2%)	51 (81%)	
nonMLR	82 (38.3%)	38 (35.5%)	18 (40.9%)	26 (41.3%)	0.7
MLR	132 (61.7%)	69 (64.5%)	26 (59.1%)	37 (58.7%)	
MLR < 2 cm	136 (63.6%)	67 (62.6%)	29 (65.9%)	40 (63.5%)	0.93
MLR > 2 cm	78 (36.4%)	40 (37.4%)	15 (34.1%)	23 (36.5%)	
no xanthoma	139 (65%)	67 (62.6%)	27 (61.4%)	45 (71.4%)	0.43
xanthoma	75 (35%)	40 (37.4%)	17 (38.6%)	18 (28.6%)	
no RE	171 (80.3%)	84 (78.5%)	37 (86%)	50 (79.4%)	0.56
RE	42 (19.7%)	23 (21.5%)	6 (14%)	13 (20.6%)	
Family history (no)	191 (89.3%)	93 (86.9%)	39 (88.6%)	59 (93.7%)	0.42
Family history (yes)	23 (10.7%)	14 (13.1%)	5 (11.4%)	4 (6.3%)	
smoke (no)	124 (57.9%)	63 (58.9%)	24 (54.5%)	37 (58.7%)	0.88
smoke (yes)	90 (42.1%)	44 (41.1%)	20 (45.5%)	26 (41.3%)	
drink (no)	120 (56.1%)	59 (55.1%)	30 (68.2%)	31 (49.2%)	0.14
drink (yes)	94 (43.9%)	48 (44.9%)	14 (31.8%)	32 (50.8%)	
age (year)	62 [56, 69]	62 [55.5, 69]	63 [56, 68.25]	62 [56.5, 68.5]	0.95
height (cm)	166.03 ± 9.03	166.32 ± 9.71	165.52 ± 8.52	165.9 ± 8.25	0.88
weight (kg)	68 [60, 80]	68 [62, 80]	65 [57.75, 80]	70 [61.5, 79.5]	0.6
BMI	24.8 [21.42, 28.6]	24.6 [21.55, 29.35]	24.95 [20.7, 28.23]	24.9 [21.55, 28.6]	0.83
Neutrophil (10^9^/L)	4.88 [2.8, 6.42]	4.34 [2.82, 5.94]	5.53 [2.85, 7.04]	5.02 [2.77, 6.05]	0.23
lymphocyte (10^9^/L)	2.27 [1.46, 3.27]	2.34 [1.6, 3.35]	2.16 [1.27, 3.06]	2.21 [1.46, 3.24]	0.62
NLR	2.03 [1.4, 2.95]	1.94 [1.23, 2.83]	2.32 [1.79, 3.87]	2.01 [1.51, 2.88]	0.09
Platelet (10^9^/L)	196 [145, 247]	196 [145, 246]	169 [134.75, 247.25]	216 [145, 251]	0.56
PLR	87.41 [61.49, 124.76]	83.71 [61.79, 117.78]	85.83 [56.13, 142.39]	89.5 [64.01, 137.42]	0.81
Hemoglobin (g/L)	132 [115, 149]	132 [114.5, 149]	128 [115, 145.25]	132 [118, 151.5]	0.69
Albumin (g/L)	49 [41, 54]	49 [41, 54]	49 [42, 56]	49 [40, 55]	0.96
HALP	67.67 [45.78, 106.16]	67.67 [49.19, 107.32]	71.62 [43.77, 109.77]	63.27 [48, 99.69]	0.86
LMR	4.9 [2.98, 8.75]	4.86 [2.98, 8.38]	5.35 [3.22, 9.95]	4.61 [2.91, 8.49]	0.96
PAR	4.05 [3.12, 5.02]	4.05 [3.11, 5.05]	3.94 [2.84, 4.83]	4.14 [3.24, 5.06]	0.43
CEA (ng/mL) Normal range [0–5]	2.6 [1.34, 3.93]	2.6 [1.35, 3.88]	2.75 [1.64, 4.36]	2.39 [1.24, 3.77]	0.33
CA19.9 (IU/mL) Normal range [0–37]	20.14 [10.62, 32.17]	19.7 [9.51, 30.84]	18.8 [10.88, 30.24]	20.9 [13.14, 32.67]	0.49
CA72.4 (IU/mL) Normal range [0–6.7]	3.49 [1.64, 5.09]	3.18 [1.53, 5.04]	3.55 [1.93, 4.79]	3.55 [1.79, 5.4]	0.73
PT (s)	11.8 [10.9, 13]	12 [11.1, 13]	11.85 [10.9, 13]	11.6 [10.7, 12.6]	0.23
AMC (10^9^/L)	0.5 [0.28, 0.64]	0.52 [0.29, 0.68]	0.42 [0.26, 0.69]	0.5 [0.29, 0.62]	0.8
RDW	13.3 [12.4, 14.1]	13.4 [12.4, 14.2]	13 [12.38, 14.03]	13.4 [12.4, 14.1]	0.7
PNI	49.01 [41.01, 54.02]	49.01 [41.01, 54.01]	49.01 [42.01, 56.01]	49.01 [40.01, 55.01]	0.96
SII	421.94 [225.28, 581.1]	370.66 [202.62, 551.35]	447.04 [272.48, 687.91]	447.36 [241.9, 597.76]	0.38
SIRI	0.92 [0.41, 1.53]	0.94 [0.38, 1.38]	0.85 [0.5, 1.67]	0.91 [0.44, 1.53]	0.86
GNRI	119.35 ± 15.61	119.66 ± 15.18	118.57 ± 15.53	119.37 ± 16.59	0.93
Atrophy range	3 [2, 4]	3 [2, 4]	3 [2.75, 4]	3 [2, 3.5]	0.21
MLRrange	2 [0, 3]	2 [0, 3]	2 [0, 3]	2 [0, 3]	0.51
Eradication time (year)	3 [2, 7]	3 [1, 6.5]	3 [2, 6]	3 [1.5, 7]	0.78

MLR, Map-Like Redness; RE: Reflux esophagitis; BMI, Body Mass Index; NLR, Neutrophil-to-Lymphocyte Ratio; PLR, Platelet-to-Lymphocyte Ratio; HALP, Hemoglobin and Albumin and Lymphocyte and Platelet score; LMR, Lymphocyte-to-Monocyte Ratio; PAR, Platelet-to-Albumin Ratio; CEA, Carcinoembryonic Antigen; CA19.9, Carbohydrate Antigen 19–9; CA72.4, Carbohydrate Antigen 72–4; PT, Prothrombin Time; AMC, Absolute Monocyte Count; RDW, Red Cell Distribution Width; PNI, Prognostic Nutritional Index; SII, Systemic Immune-Inflammation Index; SIRI, Systemic Inflammation Response Index; GNRI, Geriatric Nutritional Risk Index.

**Table 2:** Information in training cohort.

Variable	Overall	No Early Gastric Cancer	Early Gastric Cancer	Statistic	*p*_Value
Female	23 (21.5%)	18 (29%)	5 (11.1%)	3.96	0.047
Male	84 (78.5%)	44 (71%)	40 (88.9%)		
nonMLR	38 (35.5%)	37 (59.7%)	1 (2.2%)	35.12	<0.001
MLR	69 (64.5%)	25 (40.3%)	44 (97.8%)		
MLR < 2 cm	67 (62.6%)	48 (77.4%)	19 (42.2%)	12.34	<0.001
MLR > 2 cm	40 (37.4%)	14 (22.6%)	26 (57.8%)		
no xanthoma	67 (62.6%)	52 (83.9%)	15 (33.3%)	26.33	<0.001
xanthoma	40 (37.4%)	10 (16.1%)	30 (66.7%)		
no RE	84 (78.5%)	52 (83.9%)	32 (71.1%)	1.82	0.178
RE	23 (21.5%)	10 (16.1%)	13 (28.9%)		
Family history (no)	93 (86.9%)	56 (90.3%)	37 (82.2%)	0.88	0.349
Family history (yes)	14 (13.1%)	6 (9.7%)	8 (17.8%)		
smoke (no)	63 (58.9%)	39 (62.9%)	24 (53.3%)	0.63	0.427
smoke (yes)	44 (41.1%)	23 (37.1%)	21 (46.7%)		
drink (no)	59 (55.1%)	31 (50%)	28 (62.2%)	1.12	0.29
drink (yes)	48 (44.9%)	31 (50%)	17 (37.8%)		
age (year)	60.91 ± 10.59	56.69 ± 10.48	66.71 ± 7.64	−5.45	<0.001
height (cm)	166.32 ± 9.71	166.16 ± 9.74	166.53 ± 9.76	−0.19	0.846
weight (kg)	70.8 ± 13.53	72.6 ± 14.21	68.33 ± 12.25	1624.5	0.148
BMI	25.76 ± 5.46	26.42 ± 5.54	24.85 ± 5.28	1614.5	0.167
Neutrophil (10^9^/L)	4.53 ± 1.9	4.52 ± 1.94	4.56 ± 1.86	1367.5	0.865
lymphocyte (10^9^/L)	2.41 ± 0.97	2.4 ± 0.97	2.43 ± 0.98	1353.5	0.796
NLR	2.28 ± 1.59	2.26 ± 1.59	2.3 ± 1.59	1386	0.957
Platelet (10^9^/L)	198.56 ± 58.29	200.11 ± 60.19	196.42 ± 56.15	1427.5	0.84
PLR	98.14 ± 53.25	99.52 ± 54.76	96.22 ± 51.64	1414	0.907
Hemoglobin (g/L)	130.76 ± 20.63	133.26 ± 20.87	127.31 ± 20.02	1631	0.137
Albumin (g/L)	48.19 ± 7.44	48.65 ± 7.47	47.56 ± 7.43	1507.5	0.479
HALP	84.34 ± 51.67	86.52 ± 53.25	81.34 ± 49.84	1465	0.661
LMR	7.65 ± 7.86	7.54 ± 7.7	7.8 ± 8.16	1354.5	0.801
PAR	4.21 ± 1.41	4.23 ± 1.5	4.2 ± 1.28	0.09	0.93
CEA (ng/mL) Normal range [0–5]	2.64 ± 1.45	2.48 ± 1.41	2.86 ± 1.48	1167	0.151
CA19.9 (IU/mL) Normal range [0–37]	20.38 ± 11.61	20.75 ± 11.36	19.86 ± 12.04	1454	0.712
CA72.4 (IU/mL) Normal range [0–6.7]	3.35 ± 2.11	3.65 ± 2.08	3.77 ± 1.92	65.5	0.081
PT (s)	12.01 ± 1.12	12.15 ± 1.12	11.81 ± 1.12	1639	0.124
AMC (10^9^/L)	0.49 ± 0.24	0.49 ± 0.24	0.49 ± 0.25	1405	0.952
RDW	13.24 ± 1.14	13.33 ± 1.17	13.13 ± 1.09	1543.5	0.35
PNI	48.2 ± 7.44	48.66 ± 7.48	47.57 ± 7.43	1510	0.47
SII	443.9 ± 313.54	445.89 ± 298.81	441.17 ± 336.22	1413	0.912
SIRI	1.07 ± 0.88	1.07 ± 0.92	1.08 ± 0.82	1370	0.877
GNRI	119.66 ± 15.18	121.57 ± 15.31	117.04 ± 14.76	1.54	0.128
Atrophy range	3.27 ± 1.32	2.53 ± 0.86	4.29 ± 1.16	342.5	<0.001
MLRrange	1.82 ± 1.58	1.02 ± 1.29	2.93 ± 1.23	438	<0.001
Eradication time (year)	4.02 ± 3.13	3.55 ± 3.07	4.67 ± 3.13	1097.5	0.057

MLR, Map-Like Redness; RE: Reflux esophagitis; BMI, Body Mass Index; NLR, Neutrophil-to-Lymphocyte Ratio; PLR, Platelet-to-Lymphocyte Ratio; HALP, Hemoglobin and Albumin and Lymphocyte and Platelet score; LMR, Lymphocyte-to-Monocyte Ratio; PAR, Platelet-to-Albumin Ratio; CEA, Carcinoembryonic Antigen; CA19.9, Carbohydrate Antigen 19–9; CA72.4, Carbohydrate Antigen 72–4; PT, Prothrombin Time; AMC, Absolute Monocyte Count; RDW, Red Cell Distribution Width; PNI, Prognostic Nutritional Index; SII, Systemic Immune-Inflammation Index; SIRI, Systemic Inflammation Response Index; GNRI, Geriatric Nutritional Risk Index.

**Table 3:** Different machine learning’s predict performance.

Model	Test AUC	PR AUC	Accuracy	Sensitivity	Specificity	PPV	NPV
CatBoost	0.754	0.539	0.704	0.722	0.692	0.619	0.782
SVC (Poly)	0.754	0.569	0.659	0.5	0.769	0.6	0.689
LightGBM	0.741	0.534	0.659	0.722	0.615	0.565	0.761
Bagging	0.739	0.616	0.704	0.666	0.730	0.631	0.76
AdaBoost	0.737	0.619	0.704	0.666	0.730	0.631	0.76
Random Forest	0.731	0.508	0.704	0.666	0.730	0.631	0.76
Extra Trees	0.725	0.527	0.613	0.5	0.692	0.529	0.666
QDA	0.722	0.588	0.636	0.333	0.846	0.6	0.647
Gradient Boosting	0.720	0.518	0.659	0.611	0.692	0.578	0.72
SVC (RBF)	0.715	0.523	0.681	0.611	0.730	0.611	0.730
Gaussian NB	0.700	0.582	0.590	0.5	0.653	0.5	0.653
Ridge Classifier	0.698	0.502	0.590	0.5	0.653	0.5	0.653
LDA	0.698	0.502	0.613	0.611	0.615	0.523	0.695
Bernoulli NB	0.694	0.567	0.590	0.555	0.615	0.5	0.666
KNN (*k* = 3)	0.693	0.560	0.704	0.666	0.730	0.631	0.76
KNN (*k* = 5)	0.686	0.540	0.681	0.666	0.692	0.6	0.75
Logistic Regression	0.681	0.539	0.590	0.5	0.653	0.5	0.653
MLP	0.664	0.479	0.613	0.555	0.653	0.526	0.68
SGD Classifier	0.662	0.613	0.568	0.5	0.615	0.473	0.64
Decision Tree	0.632	0.660	0.636	0.611	0.653	0.55	0.708
Linear SVC	0.632	0.512	0.590	0.611	0.576	0.5	0.681

AUC, Area Under the Curve; PR AUC, Precision–Recall Area Under the Curve; PPV, Positive Predictive Value; NPV, Negative Predictive Value; SVC (Poly), Support Vector Classifier (Polynomial kernel); LightGBM, Light Gradient Boosting Machine; SVC (RBF), Support Vector Classifier (Radial Basis Function kernel); QDA, Quadratic Discriminant Analysis; LDA, Linear Discriminant Analysis; KNN, K-Nearest Neighbors; MLP, Multi-Layer Perceptron; SGD, Stochastic Gradient Descent; SVC, Support Vector Classifier.

**Table 4:** Different models’ performance.

Dataset	Model	AUC	AUC 95% CI Lower	AUC 95% CI Upper	Sensitivity	Specificity	PPV	NPV	Accuracy
Internal training	EGCM	0.943	0.900	0.987	0.888	0.919	0.888	0.919	0.906
	NLR	0.503	0.408	0.597	0	1	0	0.579	0.579
	PNI	0.541	0.446	0.635	0	1	0	0.579	0.579
	PLR	0.506	0.412	0.601	0	1	0	0.579	0.579
	SII	0.506	0.411	0.601	0	1	0	0.579	0.579
	SIRI	0.508	0.414	0.603	0	1	0	0.579	0.579
	GNRI	0.597	0.504	0.690	0.155	0.887	0.5	0.591	0.579
	HALP	0.525	0.430	0.619	0	1	0	0.579	0.579
	LMR	0.514	0.419	0.609	0	1	0	0.579	0.579
	PAR	0.486	0.391	0.580	0	1	0	0.579	0.579
Internal Test	EGCM	0.743	0.614	0.872	0.555	0.692	0.555	0.692	0.636
	NLR	0.521	0.373	0.668	0	1	0	0.590	0.590
	PNI	0.626	0.483	0.769	0	1	0	0.590	0.590
	PLR	0.478	0.331	0.626	0	1	0	0.590	0.590
	SII	0.472	0.324	0.619	0	1	0	0.590	0.590
	SIRI	0.613	0.469	0.757	0	1	0	0.590	0.590
	GNRI	0.559	0.413	0.706	0.222	0.846	0.5	0.611	0.590
	HALP	0.529	0.382	0.677	0	1	0	0.590	0.590
	LMR	0.401	0.256	0.546	0	1	0	0.590	0.590
	PAR	0.427	0.281	0.573	0	1	0	0.590	0.590
External Test	EGCM	0.905	0.832	0.977	0.76	0.894	0.826	0.85	0.841
	NLR	0.5	0.376	0.623	0	1	0	0.603	0.603
	PNI	0.502	0.378	0.625	0	1	0	0.603	0.603
	PLR	0.543	0.420	0.666	0	1	0	0.603	0.603
	SII	0.530	0.407	0.653	0	1	0	0.603	0.603
	SIRI	0.528	0.405	0.651	0	1	0	0.603	0.603
	GNRI	0.57	0.447	0.692	0.28	0.868	0.583	0.647	0.634
	HALP	0.486	0.362	0.609	0	1	0	0.603	0.603
	LMR	0.516	0.393	0.640	0	1	0	0.603	0.603
	PAR	0.517	0.393	0.640	0	1	0	0.603	0.603

EGCM, Early Gastric Cancer Model; AUC, Area Under the Curve; CI, Confidence Interval; PPV, Positive Predictive Value; NPV, Negative Predictive Value; NLR, Neutrophil-to-Lymphocyte Ratio; PNI, Prognostic Nutritional Index; PLR, Platelet-to-Lymphocyte Ratio; SII, Systemic Immune-Inflammation Index; SIRI, Systemic Inflammation Response Index; GNRI, Geriatric Nutritional Risk Index; HALP, Hemoglobin and Albumin and Lymphocyte and Platelet score; LMR, Lymphocyte-to-Monocyte Ratio; PAR, Platelet-to-Albumin Ratio.

**Table 5:** Different models’ calibration index.

Model	Brier_Train	HL_*p*_Train	Brier_Test	HL_*p*_Test	Brier_External	HL_*p*_External
EGCM	0.001	0.999	0.002	0.261	0.038	0.285
NLR	0.025	0.580	0.125	<0.001	0.118	<0.001
PNI	0.019	0.679	0.254	<0.001	0.124	<0.001
PLR	0.039	0.318	0.185	<0.001	0.119	<0.001
SII	0.027	0.500	0.325	<0.001	0.113	<0.001
SIRI	0.042	0.208	0.410	<0.001	0.124	<0.001
GNRI	0.060	0.071	0.318	<0.001	0.117	<0.001
HALP	0.026	0.574	0.408	<0.001	0.116	<0.001
LMR	0.020	0.724	0.461	<0.001	0.123	<0.001
PAR	0.067	0.133	0.303	<0.001	0.126	<0.001

EGCM, Early Gastric Cancer Model; HL, Hosmer–Lemeshow; NLR, Neutrophil-to-Lymphocyte Ratio; PNI, Prognostic Nutritional Index; PLR, Platelet-to-Lymphocyte Ratio; SII, Systemic Immune-Inflammation Index; SIRI, Systemic Inflammation Response Index; GNRI, Geriatric Nutritional Risk Index; HALP, Hemoglobin and Albumin and Lymphocyte and Platelet score; LMR, Lymphocyte-to-Monocyte Ratio; PAR, Platelet-to-Albumin Ratio.

**Table 6:** NRI and IDI in different models (EGCM as reference).

Dataset	Model	NRI	NRI_*p*	IDI	IDI_*p*	Event_NRI	Nonevent_NRI	Event_IDI	Nonevent_IDI
Internal training	NLR	−0.808	0	−0.593	0	−0.888	0.0806	−0.343	−0.249
	PNI	−0.808	0	−0.588	0	−0.888	0.0806	−0.340	−0.247
	PLR	−0.808	0	−0.592	0	−0.888	0.0806	−0.343	−0.249
	SII	−0.808	0	−0.593	0	−0.888	0.0806	−0.343	−0.249
	SIRI	−0.808	0	−0.593	0	−0.888	0.0806	−0.343	−0.249
	GNRI	−0.765	0	−0.571	0	−0.733	−0.032	−0.331	−0.240
	HALP	−0.808	0	−0.590	0	−0.888	0.0806	−0.342	−0.248
	LMR	−0.808	0	−0.592	0	−0.888	0.0806	−0.343	−0.249
	PAR	−0.808	0	−0.593	0	−0.888	0.0806	−0.343	−0.249
Internal test	NLR	−0.247	0.0768	−0.262	0.0082	−0.555	0.3077	−0.144	−0.117
	PNI	−0.247	0.0768	−0.245	0.0171	−0.555	0.3077	−0.136	−0.109
	PLR	−0.247	0.0768	−0.263	0.0082	−0.555	0.3077	−0.149	−0.114
	SII	−0.247	0.0768	−0.263	0.0079	−0.555	0.3077	−0.147	−0.115
	SIRI	−0.247	0.0768	−0.261	0.0083	−0.555	0.3077	−0.145	−0.116
	GNRI	−0.179	0.0881	−0.243	0.0251	−0.333	0.1538	−0.130	−0.113
	HALP	−0.247	0.0768	−0.257	0.0097	−0.555	0.3077	−0.143	−0.113
	LMR	−0.247	0.0768	−0.263	0.0079	−0.555	0.3077	−0.147	−0.116
	PAR	−0.247	0.0768	−0.263	0.0077	−0.555	0.3077	−0.146	−0.117
External test	NLR	−0.654	0	−0.480	0	−0.76	0.1053	−0.231	−0.249
	PNI	−0.654	0	−0.479	0	−0.76	0.1053	−0.228	−0.250
	PLR	−0.654	0	−0.477	0	−0.76	0.1053	−0.231	−0.246
	SII	−0.654	0	−0.479	0	−0.76	0.1053	−0.231	−0.248
	SIRI	−0.654	0	−0.480	0	−0.76	0.1053	−0.231	−0.249
	GNRI	−0.506	0	−0.460	0	−0.48	−0.026	−0.217	−0.242
	HALP	−0.654	0	−0.478	0	−0.76	0.1053	−0.228	−0.250
	LMR	−0.654	0	−0.482	0	−0.76	0.1053	−0.232	−0.249
	PAR	−0.654	0	−0.479	0	−0.76	0.1053	−0.231	−0.248

EGCM, Early Gastric Cancer Model; NRI, Net Reclassification Improvement; IDI, Integrated Discrimination Improvement; NLR, Neutrophil-to-Lymphocyte Ratio; PNI, Prognostic Nutritional Index; PLR, Platelet-to-Lymphocyte Ratio; SII, Systemic Immune-Inflammation Index; SIRI, Systemic Inflammation Response Index; GNRI, Geriatric Nutritional Risk Index; HALP, Hemoglobin and Albumin and Lymphocyte and Platelet score; LMR, Lymphocyte-to-Monocyte Ratio; PAR, Platelet-to-Albumin Ratio.

## Data Availability

The data that support the findings of this study are not publicly available due to patient privacy and institutional data sharing policies. However, de-identified data and code for model development are available from the corresponding author (wangwenfj@163.com) upon reasonable request.
